# A new species of *Rangabradya* (Copepoda, Harpacticoida, Ectinosomatidae) from a cave in Satun Province, southern Thailand

**DOI:** 10.3897/zookeys.1009.54554

**Published:** 2021-01-04

**Authors:** Chaichat Boonyanusith, Sujeephon Athibai

**Affiliations:** 1 School of Biology, Faculty of Science and Technology, Nakhon Ratchasima Rajabhat University, Nakhon Ratchasima 30000, Thailand Nakhon Ratchasima Rajabhat University Nakhon Ratchasima Thailand; 2 Applied Taxonomic Research Center and Department of Biology, Faculty of Science, Khon Kaen University, Khon Kaen 40002, Thailand Khon Kaen University Khon Kaen Thailand

**Keywords:** Anchialine caves, cave-dwelling copepods, Satun Geopark, Satun Province, Southeast Asia

## Abstract

A representative of the family Ectinosomatidae was discovered in a temporary pool in a cave in the Satun Province, southern Thailand. Based on the characteristics of the antennary exopod, armature of the female fifth leg, and configuration of the male sixth leg, a new species of the genus *Rangabradya* was identified, representing the first record of the genus in the continental waters of Thailand and in Southeast Asia. The new species can be distinguished from *Rangabradya indica* by the configurations of the fifth and the sixth legs in both sexes, the body ornamentation of the urosomite, and the armature of the mouthparts. These structures show a more primitive state in the new species. Accordingly, a new subgeneric rank in the genus *Rangabradya*, *Siamorangabradya***subgen. nov**, was established to accommodate the Thai species and Rangabradya (Siamorangabradya) wongkamhaengae**subgen. et sp. nov.** was described. Also, the key to all 23 genera of the family was updated.

## Introduction

**Figure 1.**
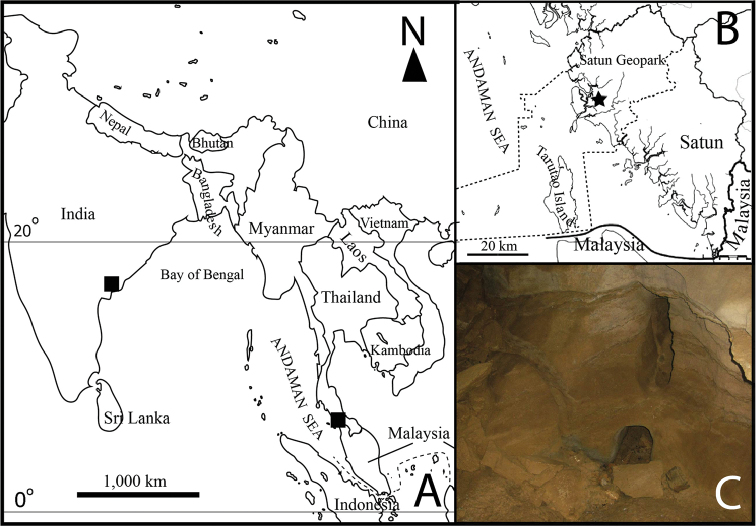
Geographical location and details of a sampling site **A** distribution of the representatives of the genus *Rangabradya* (black squares) **B** location of the Khay Cave in Satun Province, Thailand (star) **C** sampling point in the cave.

Satun Province is located in southern Thailand and is part of the continental area of the Sunda Subregion (Fig. 1A). Evidence for an Australian affinity of Late Cambrian fossils on Tarutao Island (a part of the province in the Andaman Sea) has suggested an eastern Gondwana origin of the Sibumasu Terrane (Shergold et al. 1988; Metcalfe 1998). A karst topography dominates the north and east of the province characterized by limestone mountains with sharp blade-like tips, sinkholes, caves, and a network of underground water channels. The west of the province is represented by a lowland area covered by sediments of the ancient shallow sea. A rich diversity of fauna endemic to the country is expected for Satun Province, where several new taxa have recently been discovered from karstic areas (e.g., Sumontha et al. 2014; Liu et al. 2017; Nilsai et al. 2017; Promdam et al. 2017). To date, two new copepod species from this province have been described, namely, *Onychocamptus satunensis* Boonyanusith, Saetang, Wongkamhaeng & Maiphae, 2018 and *Boholina laorsriae* Boonyanusith, Wongkamhaeng & Athibai, 2020 (Boonyanusith et al. 2018, 2020). The latter is the first record of the subterranean Calanoida in Thailand.

The family Ectinosomatidae Sars, 1903 is speciose, encompassing 325 valid species from 23 genera (George and Schwabe 2019). Representatives of the family occupy a wide array of habitats. Most species are known from the fine sediments of the sublittoral marine environments (Kihara and Huys 2009), although, many species inhabit the abyssal plains (Bodin 1968; Seifried et al. 2007), and a few have been described from the surface and subterranean freshwater habitats (Karanovic and Pesce 2001). Additionally, a few species are symbionts (Boxshall and Halsey 2004; George and Schwabe 2019).

Among the genera currently known as members of the family Ectinosomatidae, the genus *Rangabradya* Karanovic & Pesce, 2001 is a subterranean freshwater representative. The only known species of the genus is *Rangabradya indica* Karanovic & Pesce, 2001, previously described from a freshwater bore-well in India (Fig. 1A). Morphologically, the genus is distinguished from all other Ectinosomatidae by the presence of a single seta on the endopodal lobe of the male fifth leg and the characteristics of the antennary exopod (Karanovic and Pesce 2001).

Five years ago, a representative of the family Ectinosomatidae was encountered in samples of cave-dwelling copepods from a cave in the La-Ngu District of Satun Province, southern Thailand. The morphology of body shape, maxilla, and swimming legs indicates the close relatedness of the Thai specimens and the genus *Rangabradya*, but a detailed examination revealed a more primitive state in the mouthparts, fifth and sixth swimming legs, and ornamentation of the urosomites. However, there is no apomorphic character to distinguish it as a new genus. For this reason, a new subgeneric rank was created within the genus *Rangabradya*, and the new cave-dwelling Harpacticoida from southern Thailand was named Rangabradya (Siamorangabradya) wongkamhaengae subgen. et sp. nov. In this paper, descriptions and illustrations of the new species are presented. Furthermore, an updated key to all 23 genera of the family is provided.

## Materials and methods

Samples were collected from a temporary pool in Khay Cave of Satun Province, southern Thailand, using a hand net with a mesh size of 60 µm, and stored in a solution of 4% formaldehyde. Specimens were sorted under a stereomicroscope and stored in 70% ethanol. Before the morphological examination, the specimens were placed in a mixture of glycerol and 70% ethanol (ratio ~1:10 v/v) for 30 minutes. They were subsequently completely dissected and mounted on slides in glycerol and covered with coverslips.

The examination of body parts and ornamentations was performed under a Nikon ECLIPSE E200 compound light microscope at 1000× magnification. The habitus and body appendages were drawn using a drawing tube attached to the compound microscope, and the final versions of the illustrations were prepared in Adobe Illustrator CC 2020. For a detailed examination, additional material was dehydrated in ethanol in a series of concentrations increasing from 70% to 100% and dried in a critical point dryer. Afterwards, they were mounted on stubs, coated with gold, and examined using a scanning electron microscope (Leo 1450VP).

Morphological descriptions were made following the terminology used in Huys and Boxshall (1991). The terms ‘pars incisiva’ and ‘lacinia’ were retained for the descriptions of the mandible. The following descriptive abbreviations are used in the description and figures:

**Endp** endopod;

**Exp** exopod;

**Endp/Exp-1 (2, 3)** proximal (middle, distal) segment of endopod and exopod;

**ae** aesthetasc;

**I** spine;

**P1–P6** swimming legs 1–6.

The type material has been deposited at the Princess Maha Chakri Sirindhorn Natural History Museum, Prince of Songkla University, Songkhla, Thailand (**PSUNHM**).

## Taxonomy

**Order Harpacticoida Sars, 1903**

**Family Ectinosomatidae Sars, 1903**

**Genus *Rangabradya* Karanovic & Pesce, 2001**

### Subgenus Siamorangabradya subgen. nov.

Taxon classificationAnimaliaEctinosomatidaeEctinosomatidae

B7C8A964-F280-50AE-9D12-19175ABE530A

http://zoobank.org/33D0FA48-E799-409E-84B0-F546BED6BC2A

#### Diagnosis.

Ectinosomatidae, with fusiform habitus. Urosomite with spinule ornamentation. Antennule six-segmented in both male and female; third segment longest. Distal segment of antennary Exp elongate, with two apical setae; longest seta normally developed; shortest one as long as segment bearing it. Mandible with additional spine at dorsal site of pars incisiva. Coxa of maxillule free. Maxilla straight, with three setae on distal endite of syncoxa and allobasis with two medial setae. Maxilliped three-segmented, with four setae on Endp. Armature formula of Exp-3, from P1–P4: 5.6.6.6. Female P5Exp partly fused with baseoendopod, with three marginal setae; endopodal lobe of baseoendopod with two apical elements. Female P6 reduced to small protuberance with one apical seta on peduncle. Male P5Exp completely fused to baseoendopod, with two apical setae on endopodal lobe. Male P6 reduced to simple plate and unarmed.

#### Type species.

Rangabradya (Siamorangabradya) wongkamhaengae subgen. et sp. nov.

#### Etymology.

The subgenus is named after Siam, Thailand’s former name, prefixed to the existing generic name *Rangabradya*. The name is a feminine noun in the nominative singular.

### Rangabradya (Siamorangabradya) wongkamhaengae sp. nov.

Taxon classificationAnimaliaEctinosomatidaeEctinosomatidae

3DCD9522-D643-59B8-B177-F418789876B3

http://zoobank.org/17517BF1-EA91-46A4-98C5-66F8144065DF

**Figure 2.**
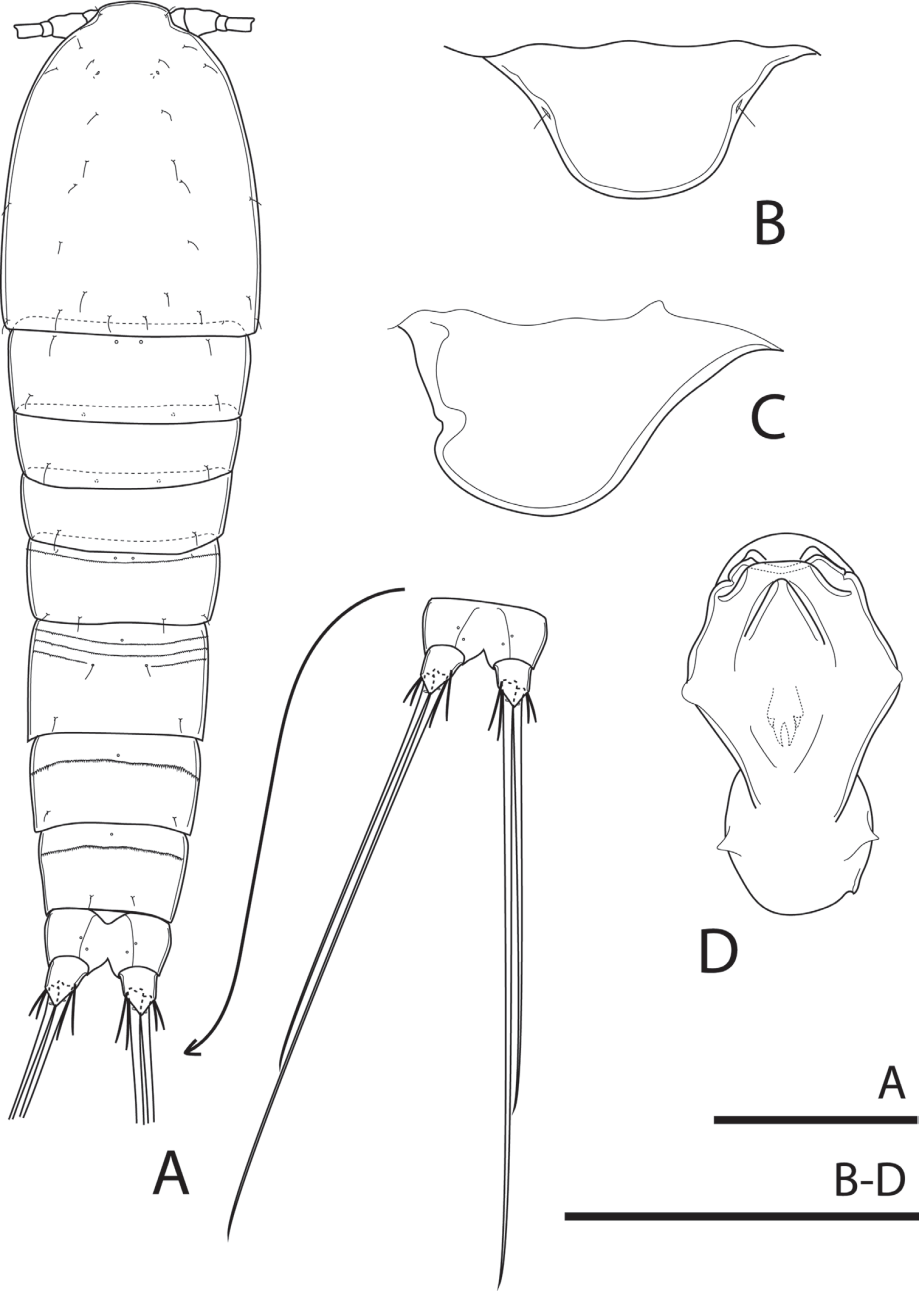
Rangabradya (Siamorangabradya) wongkamhaengae subgen. et sp. nov. female **A** habitus, dorsal view **B** rostrum, frontal view **C** labrum, lateral view **D** labrum, ventral view. Scale bars: 100 μm (**A**); 50 μm (**B−D**).

**Figure 3.**
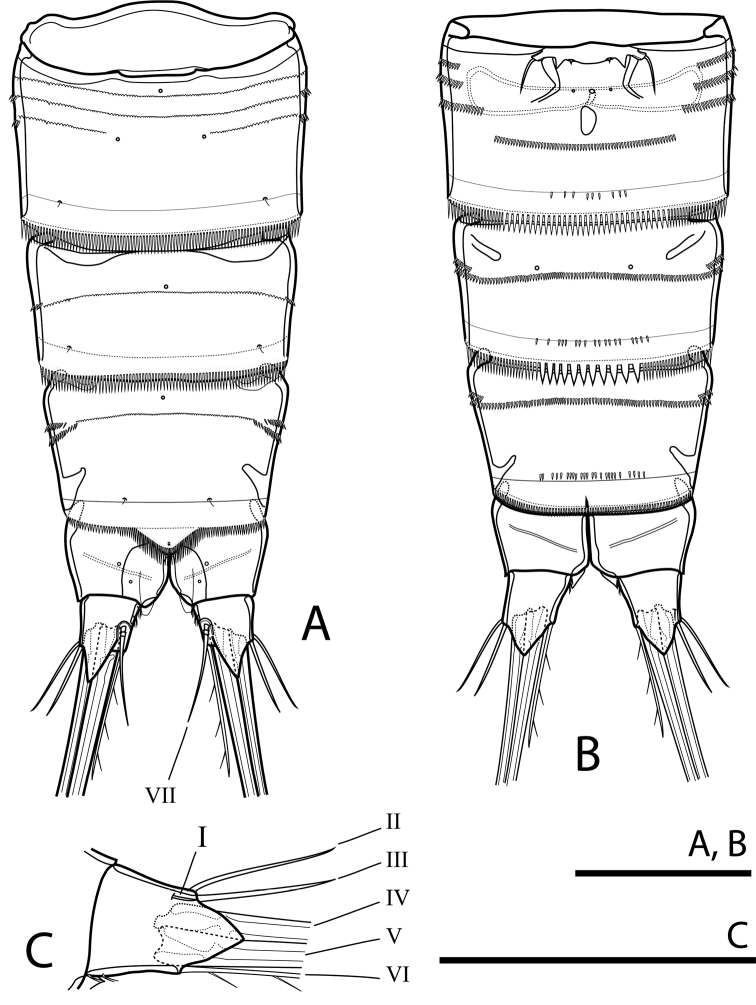
Rangabradya (Siamorangabradya) wongkamhaengae subgen. et sp. nov. female **A** urosome, dorsal view **B** urosome, ventral view **C** caudal ramus, ventral view. Scale bars: 50 μm. Roman numerals correspond to setal number.

**Figure 4.**
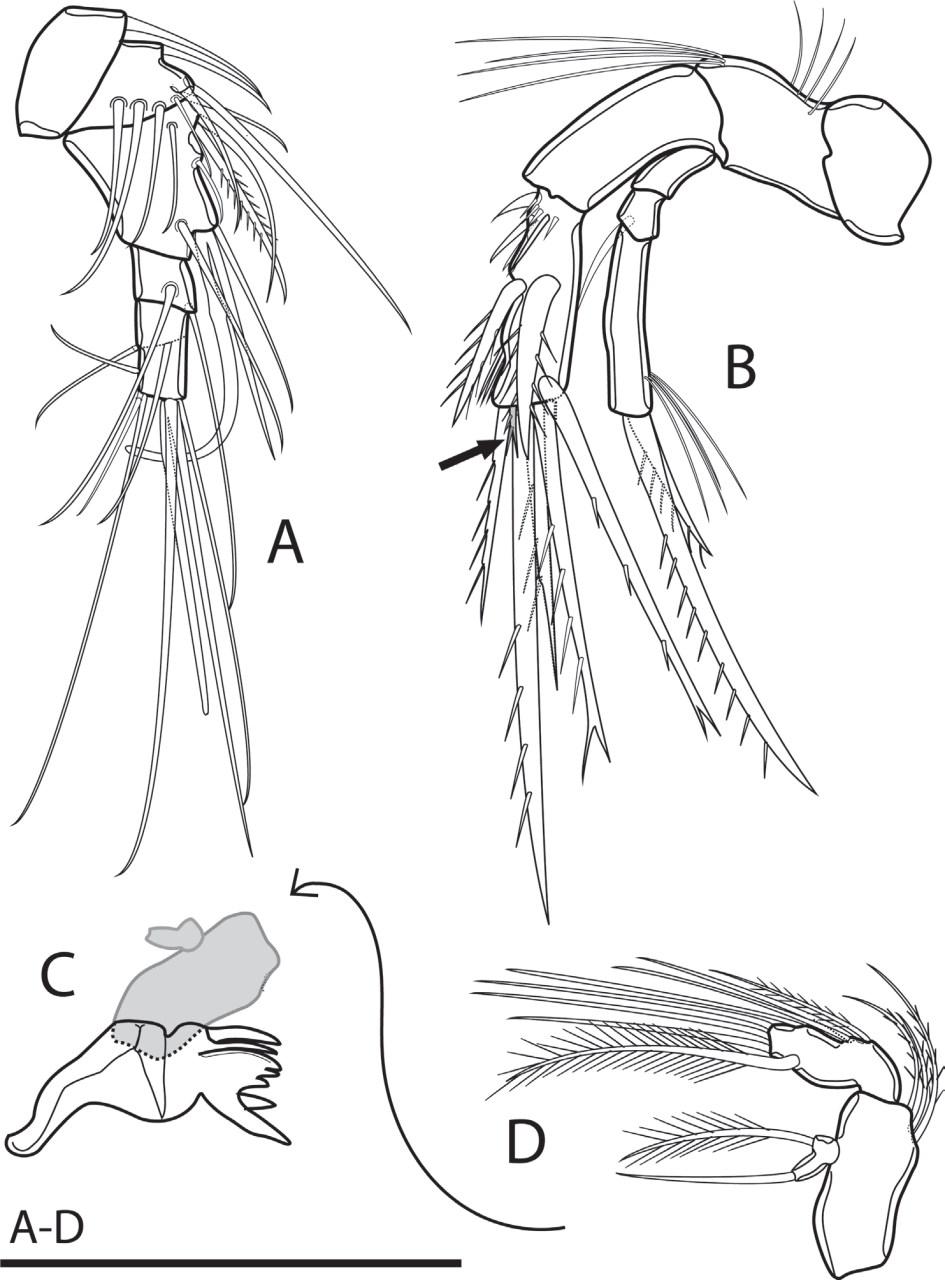
Rangabradya (Siamorangabradya) wongkamhaengae subgen. et sp. nov. female **A** antennule **B** antenna **C** praecoxa of mandible **D** mandibular pulp. Scale bars: 50 μm.

**Figure 5.**
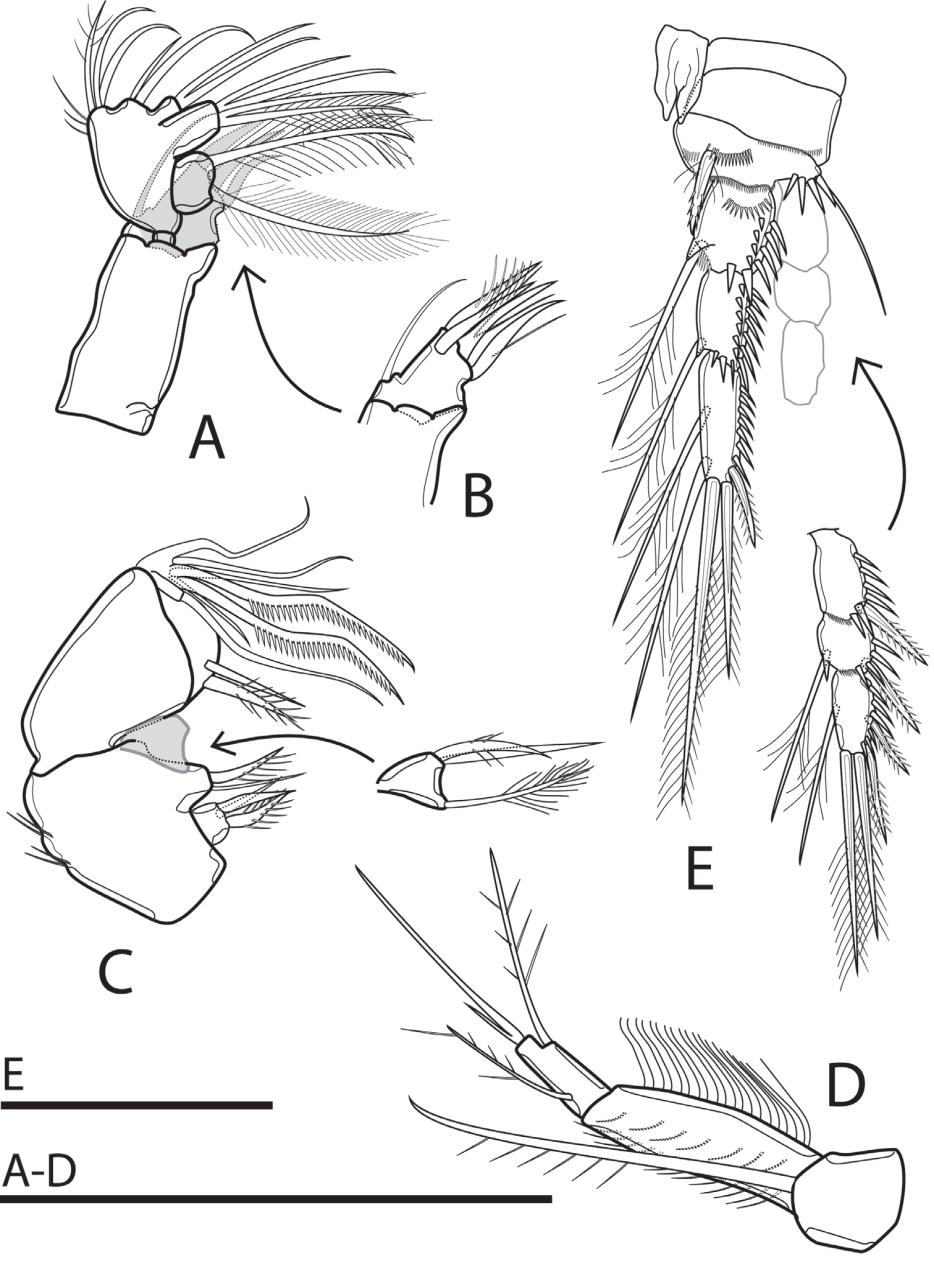
Rangabradya (Siamorangabradya) wongkamhaengae subgen. et sp. nov. female **A** maxillule **B** maxillular praecoxal arthrite **C** maxilla **D** maxilliped **E**P1. Scale bars: 50 μm.

**Figure 6.**
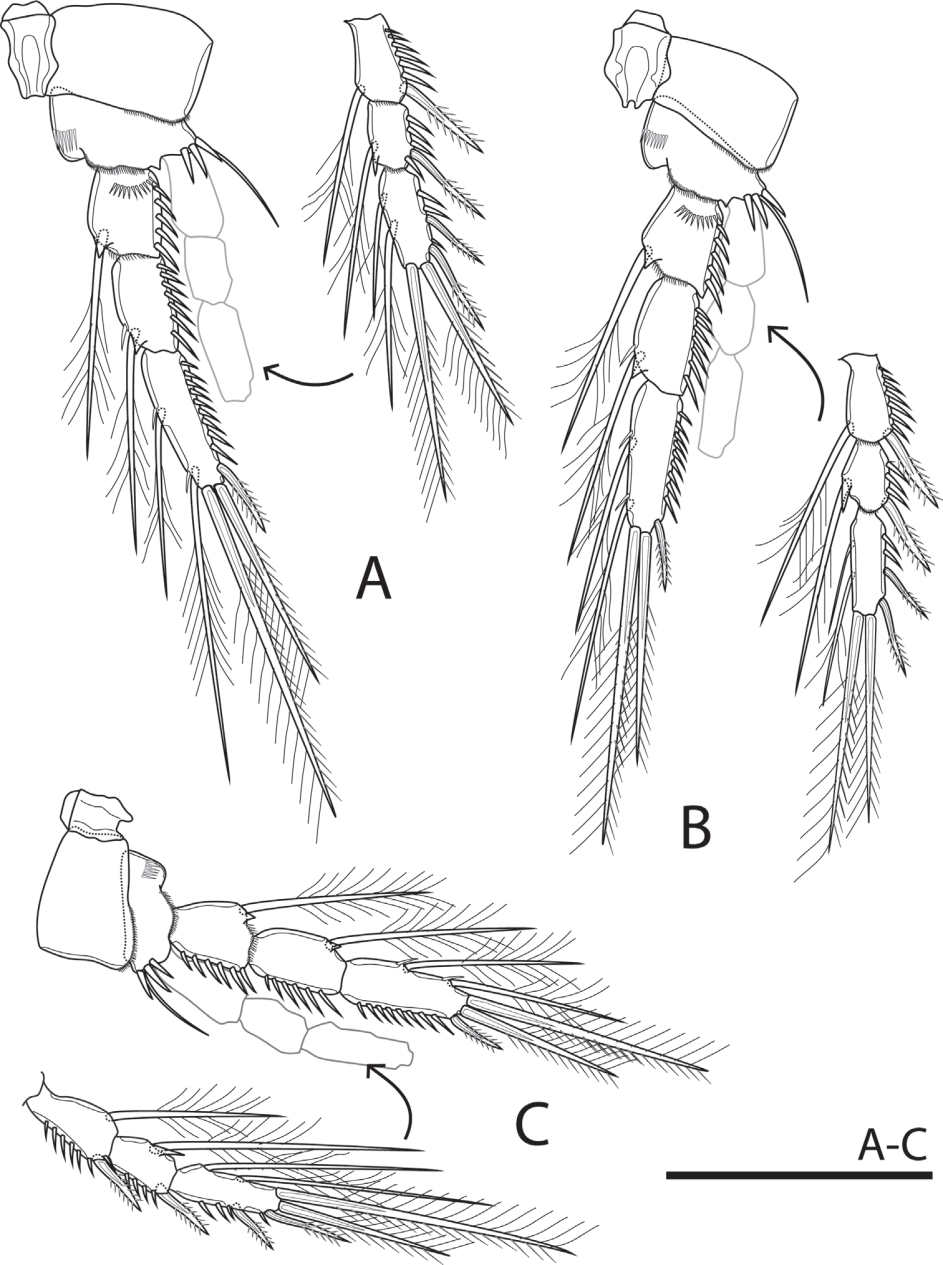
Rangabradya (Siamorangabradya) wongkamhaengae subgen. et sp. nov. female: **A**P2**B**P3**C**P4. Scale bars: 50 μm.

**Figure 7.**
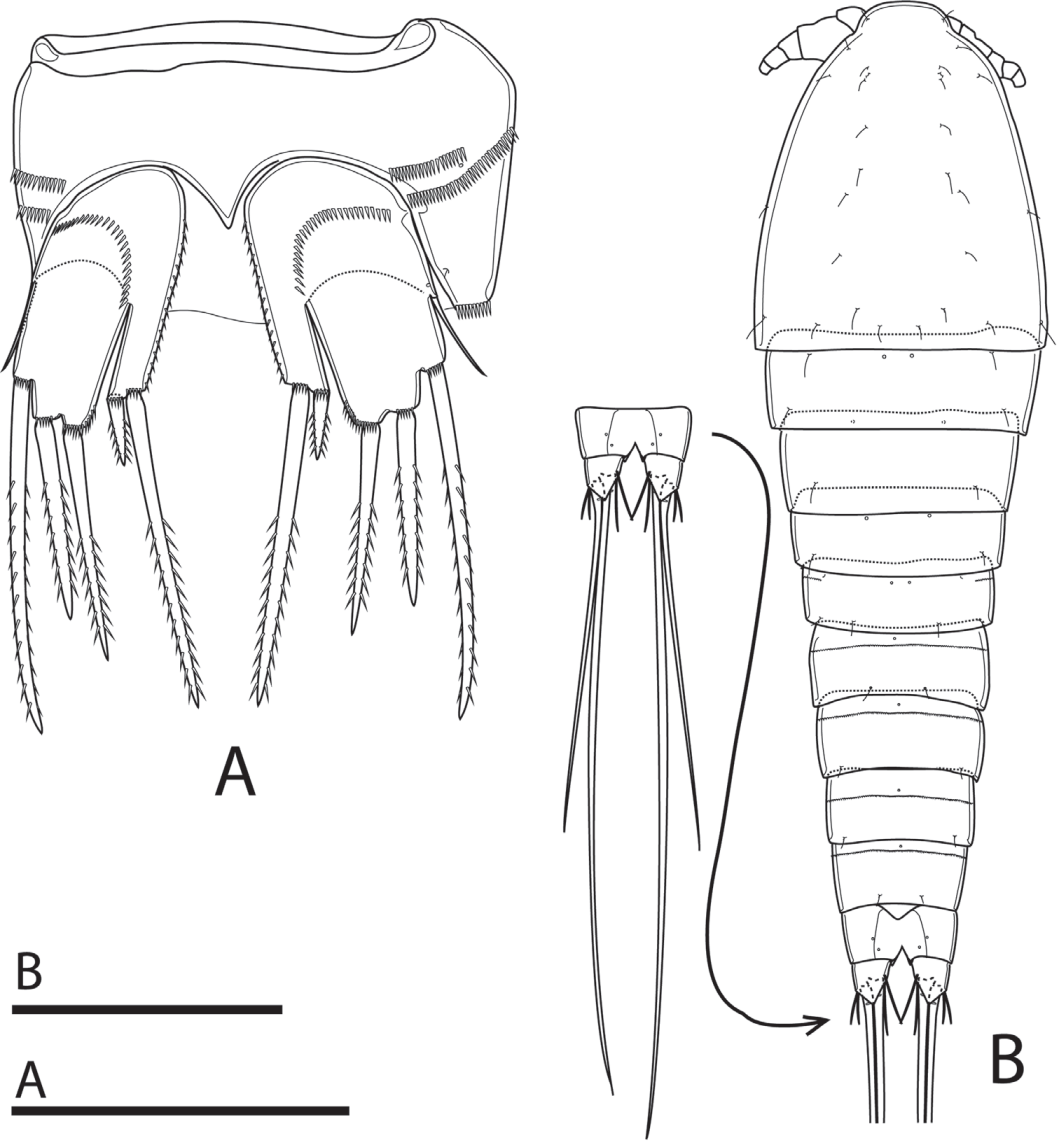
Rangabradya (Siamorangabradya) wongkamhaengae subgen. et sp. nov. female **A**P5; male: **B** habitus. Scale bars: 50 μm (**A**); 100 μm (**B**).

Figures 2 , 3 , 4 , 5 , 6 , 7A , 9 (female); 7B, 8, 10 (male)

#### Material examined.

***Holotype***: Thailand • 1 ♀ (adult), 485 μm long; Satun Province, Khay Cave; 6°53'40"N, 99°46'44"E, 17 m a.s.l.; 17 Dec. 2014; C. Boonyanusith leg.; hand net; completely dissected and mounted on a slide in glycerol and sealed with nail polish; PSUZC-PK2005-01. ***Allotype***: Thailand • 1 ♂ (adult), 428 μm long, collection data as for holotype; PSUZC-PK2005-02. ***Paratypes***: Thailand • 1 ♀ (adult) and 1 ♂ (adult); same data as for holotype; PSUZC-PK2005-03 and PSUZC-PK2005-04, respectively.

#### Additional material.

Thailand • 3 ♂♂ (adult), 1 ♀ (adult), 3 copepodids; same data as for holotype; preserved in 70% ethanol; retained in collection of the first author (CB).

#### Type locality.

Khay Cave in La-Ngu District, Satun Province, Thailand. The cave’s geography and morphology have previously been described in Boonyanusith et al. (2020) (Fig. 1).

#### Description of female.

Total body length, excluding caudal setae, 485–489 µm (mean = 487; *N* = 3). Preserved specimens colourless. Habitus fusiform, gradually tapering posteriorly, with maximum width at posterior margin of cephalothorax (Figs 2A, 9A). Rostrum (Fig. 2B) well developed, broadly rounded, completely fused with cephalothorax, with two lateral sensilla near base. Prosome ca 1.6× as long as urosome (including caudal rami), comprising cephalothorax and three free pedigerous somites. Cephalothorax ca 1.1× as long as wide and ca 0.5× as long as length of prosome; posterior margin smooth. Distribution of sensilla and cuticular pores as illustrated (Figs 2A, 9B). All free pedigerous somites dorsally with a pair of pores on anterior margin; finely spinulated hyaline frill incised on posterior margin.

Urosome comprising fifth pedigerous somite, genital double-somite, and three free abdominal somites (Fig. 2A). Posterior margin of all urosomites with finely spinulated hyaline frill, except anal somite; hyaline frill on genital double-somite and subsequent somite uniform in width dorsally and laterally, and central part wider than outer parts ventrally (Fig. 3A, B). Ornamentation as illustrated (Figs 2A, 3A, B). Fifth pedigerous somite wider than long, dorsally with pair of cuticular pores near anterior margin plus continuous row of minute spinules and four sensilla along posterior margin (Fig. 2A). Two rows of spinules lateroventrally, positioned next to implantation of P5, with one pore close to tip of proximal row (Figs 7A, 9G). Genital double-somite slightly wider than long, with three pores dorsally, arranged in triangle. Two sensilla near posterior margin and three rows of minute spinules dorsally on anterior half of double-somite; distal row with short gap medially (Fig. 3B). Ventrally a pore on either side of copulatory pore and two transverse rows of minute spinules along medial margin. Antero-laterally with three additional rows of relatively strong spinules, continuing into rows of spinules dorsally. Genital field (Fig. 3B) with small copulatory pore medially, at 1/3 of the double-somite length. Short seta representing P6 on peduncle. Second and third abdominal somites, with one single pore mid-dorsally, near anterior margin plus pair of sensilla dorsally, near posterior margin and row of minute spinules, next to cuticular pore. Ventrally pair of cuticular pores present in second abdominal segment, but absent in the subsequent one, with lateral and lateroventral row of spinules anteriorly. Additional row of spinules ventrally, near posterior margin. Third abdominal somite with cuticular bell-shaped flap dorsally, representing pseudoperculum. Anal somite with cleft medially, with two pairs of cuticular pores dorsally.

Caudal rami (Figs 2A, 3A, C) slightly divergent, as long as anal somite, ca 1.3× as long as wide, with transparent triangular lappets dorsally and ventrally, reaching beyond fracture plane of inner apical seta (V); each ramus armed with seven setae. Lateral accessory seta (I) minute (Figs 3A, 10D). Lateral seta (II) and outermost apical seta (III) subequal in length. Outer apical seta (IV) and inner apical seta (V) well developed, with fracture plane; seta V longest, ca 1.3× as long as seta IV. Innermost apical seta (VI) ornamented with spinules on inner margin, ca 1.5× as long as seta II. Dorsal seta (VII) biarticulate, inserted at ½ length of ramus on medial margin, ca 1.5× as long as seta II.

Antennule (Figs 4A, 9C) short, six-segmented. Third segment longest, with robust and curved aesthetasc, reaching well beyond tip of antennule. Ultimate segment incompletely separated from preceding one, with remnant of ancestral articulation and aesthetasc on tip of segment. Both aesthetascs combined as acrothec (common base of seta and aestetasc). Armature formula: 1-[1], 2-[8], 3-[5 + 1 pinnate + (1 + ae)], 4-[2], 5-[2], 6-[5 + (1 + ae)].

Antenna (Figs 4B, 9D, E) biramous. Coxa short, without ornamentation. Basis with transverse row of long spinules at ½ length of medial margin and set of long setules on distal medial corner. Exp three-segmented. Exp-1 bare, Exp-2 ca 0.5× as long as Exp-1, with seta subapically. Exp-3 elongate, as long as Endp-2, ca ⅗ of length of Exp, with two apical setae; shorter one as long as segment bearing it, longer one ca 2.2× as long as shorter one and ca 2× as long as segment bearing it. Row of long setules subapically. Endp two-segmented. Endp-1 bare. Endp-2 with short seta accompanied by two groups of strong spinules on proximal lateral corner. Two strong spiniform setae at ½ length of Endp-2, and six spiniform apical setae, one very short. All spiniform setae on Endp-2 robust, with spinules along margins.

Labrum (Fig. 2D) large, rhomboidal in ventral view, strongly bulging, without beak on posterior margin.

Mandible (Figs 4C, D, 9E) comprising sclerotized coxal gnathobase and mandibular palp. Coxal gnathobase with two spines at ventral base. One spine at dorsal site of tridentate pars incisiva; lacinia unidentate. Mandibular palp comprising basis, one-segmented Exp and one-segmented Endp. Basis large, armed with three setae on distal medial corner. Exp small, armed with lateral and apical seta; lateral seta plumose. Endp armed with four setae laterally. Three setae apically, and one robust, plumose seta subapically.

Maxillule (Figs 5A, B, 9E, F) with large praecoxa. Praecoxal arthrite mobile, bearing slender seta at base plus three spines apically and one spine subapically. Anterior apical and subapical spines on praecoxal arthrite with row of curved long spinules, two other spines armed with few setules. Coxa small, one-segmented, bare. Basis and Endp completely fused, with two endites and endopodal lobe. Proximal endite with three subequal setae; distal endite with two setae. Endopodal lobe with two lateral and four apical setae, each seta fused with neighbouring one at its base. Exp free, one-segmented, with two plumose setae.

Maxilla (Figs 5C, 9F) non-prehensile, with slight angle between syncoxa and allobasis. Syncoxa with rows of spinules laterally and three endites along medial margin. Proximal endite at ⅓ of syncoxa length, with three setae apically; one robust and pinnate, other two smooth. Middle endite situated at ½ length of syncoxa, with one robust, pinnate seta and one smooth, slender seta apically. Distal endite triangular, mobile, with three elements; one plumose, one smooth, and one curved and pinnate. Medial margin of allobasis with one bare and one pinnate seta, at ½ length of segment. Distal endite small, with long smooth seta apically. Endp short, inconspicuous, with four bare setae, unequal in length and two robust, uni-pinnate setae.

Maxilliped (Fig. 5D) three-segmented, comprising syncoxa, basis, and one-segmented Endp. Syncoxa with one long, robust, uni-pinnate seta at distal medial corner. Basis long, with two rows of long spinules anteriorly and one row of long setules on outer margin. Endp with seta at ⅓ of segment length, with bipinnate seta subapically. Two setae apically fused at base, longer one ca 4× as long as shorter one.

P1–P4 with three-segmented Exp and Endp. Endp always longer than Exp. Segments with rows of strong spinules on outer margins and without ornamentations on inner margins. Armature formula as follows (legend: inner-outer element; inner-apical-outer; Arabic numerals representing setae; Roman numerals representing spines):

**Table:** 

Legs	Basis	Exopod			Endopod		
		1	2	3	1	2	3
P1	I-1	0-I	1-I	1-II-II	1-0	1-0	2-II-I
P2	0-1	1-I	1-I	2-II-II	1-0	1-0	2-II-I
P3	0-1	1-I	1-I	2-II-II	1-0	1-0	2-II-I
P4	0-1	1-I	1-I	2-II-II	1-0	1-0	2-II-I

P1 (Fig. 5E). Coxa rectangular, with continuous row of spinules at distal lateral corner on anterior surface. Intercoxal sclerite narrow, elongate, free margin with obtuse lobes, without surface ornamentation. Basis with row of spinules at base of insertion of inner spine. Row of three or four spinules on distal lateral corner, near insertion of outer seta. Exp reaching just midlength of Endp-3. Endp-1 with additional row of spinules on anterior surface.

P2–P4 (Fig. 6A–C) with coxa and intercoxal sclerite similar to those of P1. Basis with short row of spinules on inner margin and row of strong spinules on distal lateral corner. Exp and Endp as in P1.

P5 (Figs 7A, 9G). Baseoendopodal lobe well developed, slightly shorter than Exp. Left and right leg distinctly separated. Endopodal lobe with two pinnate, spiniform spines; outer one short, inner one ca 6× as long as outer one. Smooth, slender seta on outer margin of basis, with cuticular pore near insertion of seta. Anterior surface of baseoendopod with arched row of spinules. Exp partly fused with baseoendopod, without surface seta and with three robust pinnate spines; outermost one longest, as long as inner spine on endopodal lobe. Inner two spines as long as Exp, subequal in length.

P6 (Fig. 3B) reduced to minute prominence, forming simple plate near anterior margin of genital double-somite. With one short seta on peduncle on each side of plate.

#### Description of male.

Body slightly smaller than in female, fusiform. Total body length, excluding caudal setae, 427–431 µm (mean = 429; *N* = 3). Preserved specimens colourless. Prosome ca 1.5× as long as urosome (Fig. 7B). Cephalothorax longer than wide, ca 0.5× as long as length of prosome. Rostrum and surface ornamentation of prosome as in female.

**Figure 8.**
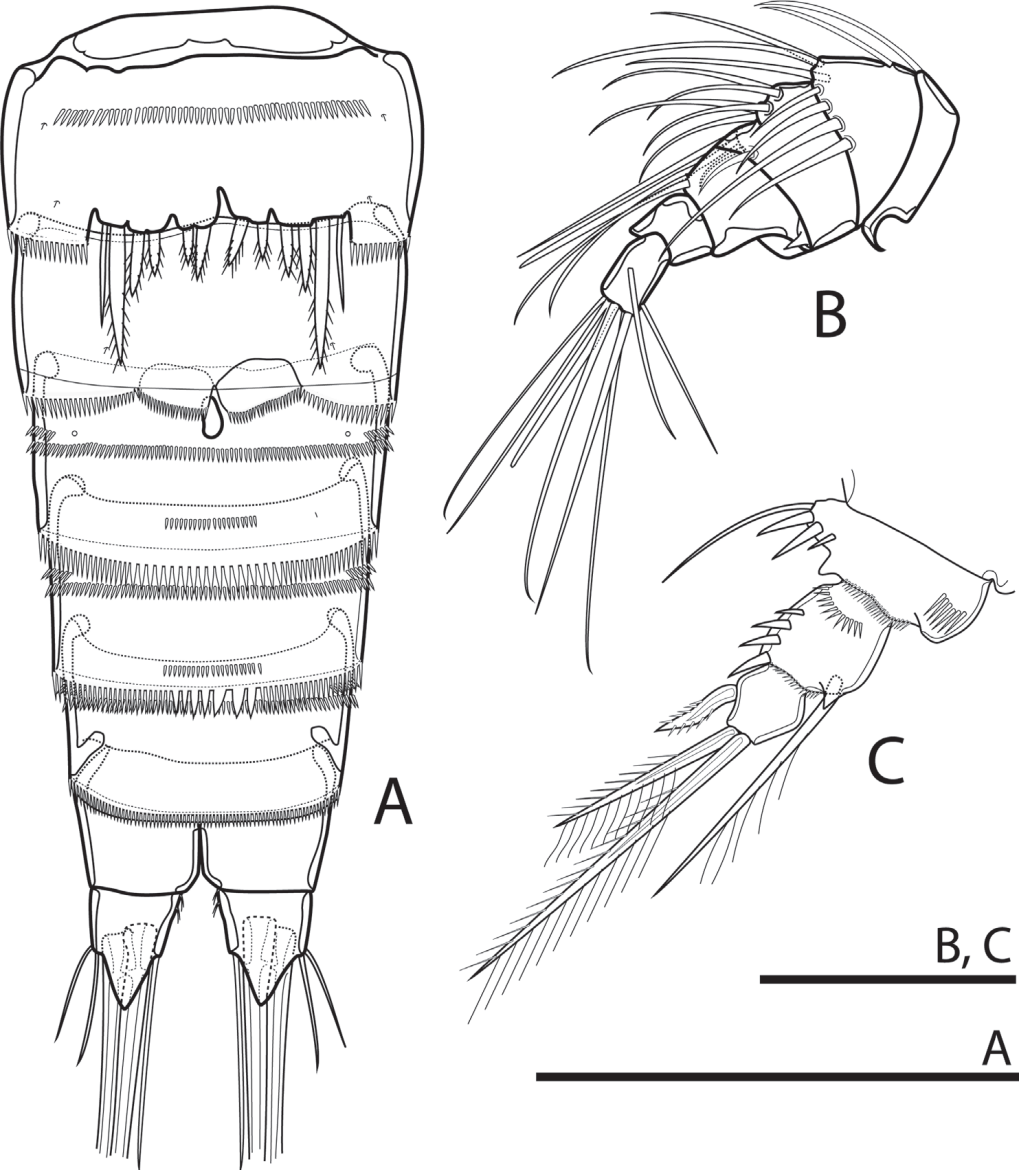
Rangabradya (Siamorangabradya) wongkamhaengae subgen. et sp. nov. male: **A** urosome, ventral view **B** antennule **C** endopod of P2. Scale bars: 50 μm.

Urosome (Figs 7B, 8A) five-segmented, comprising fifth pedigerous somite, genital somite and four free abdominal somites. All somites with finely spinulated hyaline frill on posterior margin, except anal somite. Fifth pedigerous somite as in female. Genital somite with continuous row of minute spinules dorsally and row of strong spinules ventrally. Cuticular pore near anterior margin mid-dorsally and pair of sensilla near posterior margin. Genital field with copulatory pore mid-ventrally. First to third abdominal somites each with row of minute spinules dorsally, continuing into row of strong spinules ventrally. Sixth thoracic somite with finely spinulated hyaline frill on posterior margin. Ornamentation of first abdominal to anal somites as in female.

Antennule (Fig. 8B) geniculate, six-segmented, with geniculated articulation between fourth and fifth segment. Third segment with apical aesthetasc on peduncle. Armature formula 1-[1], 2-[8], 3-[7 + (1 + ae)], 4-[2], 5-[0], 6-[7 + (1 + ae)]. Aesthetasc on third segment robust, reaching well beyond tip of antennule.

Rostrum, antenna, mouthparts and P1–P4 (not figured) as in female.

P5 (Fig. 8A). with simple plate fused to somite bearing it. Left and right leg separated by cleft. Exp completely fused to baseoendopod. Smooth soft seta on outer margin of basis. Endopodal lobe with two pinnate spiniform setae apically. Inner one slightly longer than outer one. Exopodal lobe with three pinnate spiniform setae. Outermost one longest, ca 2× as long as median one, and ca 3× as long as innermost one.

P6 (Fig. 8A) reduced to simple, rectangular plate, without armature and ornamentation. Posterior margin serrated. Left leg distinct, but right leg fused to somite bearing it.

#### Variability.

No variability was observed in the female. In one male, on the right P3Endp were two segments only. The terminal segment short, armed with one lateral spine and two apical setae, unequal in length (Fig. 8C). In all specimens of the male, left and right P6 are asymmetrical. The left P6 is articulated, but the right P6 is completely fused to genital somite.

#### Etymology.

The species name is a feminine noun in the genitive singular case, named after Dr Koraon Wongkamhaeng (Kasetsart University) in honour of her contribution to the research into the diversity of cave-dwelling copepods in Satun and Songkhla provinces.

#### Distribution.

The species is known only from the type locality.

#### Ecology.

As previously mentioned in the descriptions of *Boholina laorsriae* (Boonyanusith et al. 2020), the new species was collected from a temporary pool, where there is a small opening on the cave wall (Fig. 1C). The water temperature was 24.6 °C, pH 8.93, electrical conductivity 450 µS cm^-1^, dissolved oxygen 5.7 mg L^-1^, and salinity 0.2 ppt. The new species was accompanied by other species, including *Thermocyclops crassus* (Fischer, 1953), *Boholina laorsriae*, *Metacyclops* sp., *Mesocyclops* sp., and *Schizopera* sp.

**Figure 9.**
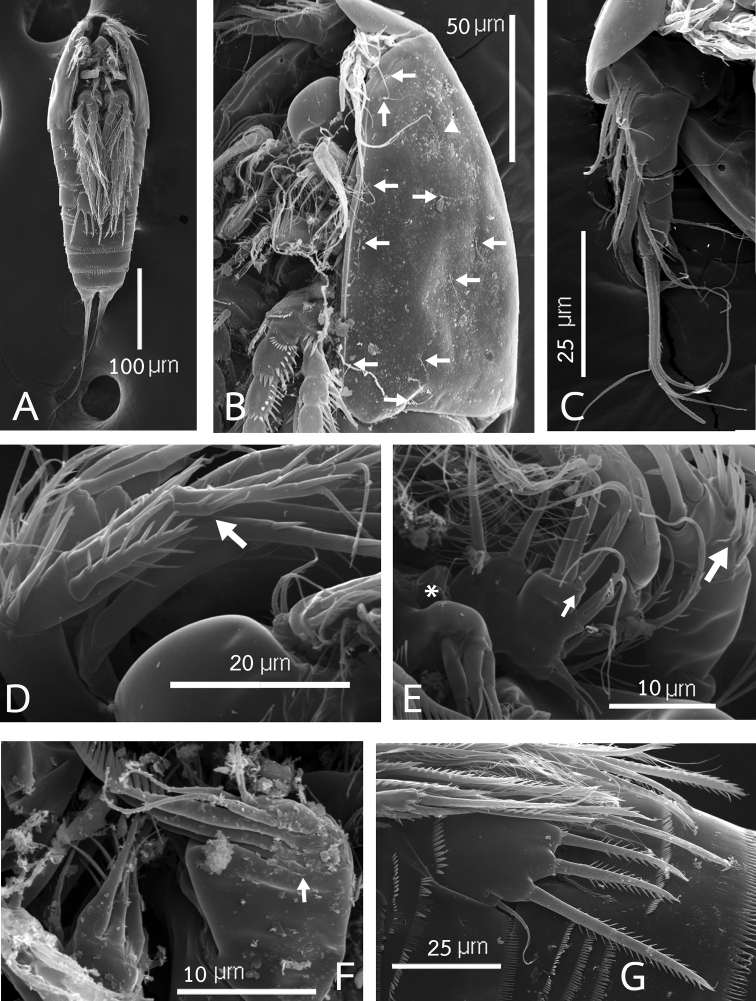
Rangabradya (Siamorangabradya) wongkamhaengae subgen. et sp. nov. Scanning electron microscope photographs **A** female habitus, ventral view **B** cephalothorax, ventrolateral view **C** antennule **D** distal segment of antenna **E** maxillule **F** maxilla **G**P5. The arrows and the triangular in figure B indicate the sensilla and integumental pore, respectively. The asterisk in figure **E** indicates coxa of the maxillule.

**Figure 10.**
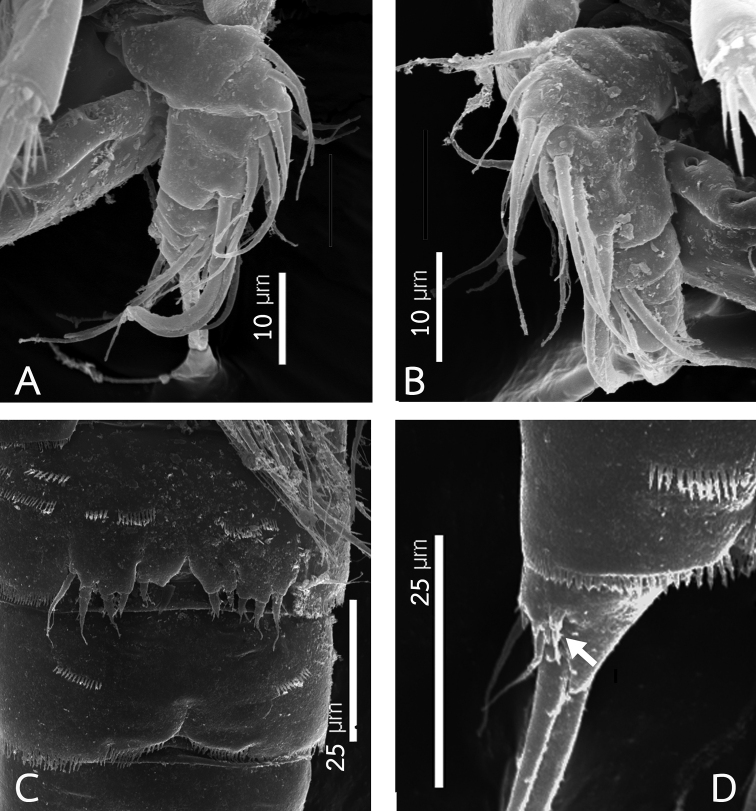
Rangabradya (Siamorangabradya) wongkamhaengae subgen. et sp. nov. Scanning electron microscope photographs, male **A** right antennule **B** left antennule **C**P5 and P6 of copepodid 5 **D** caudal ramus of copepodid 5.

## Discussion

Two characters commonly used in assessing phylogenetic relationships of the family Ectinosomatidae are: 1) the shapes of the cephalothorax and habitus and 2) the orientation of the maxillary allobasis.

The recently known genera of the family can be divided into three groups, according to the shapes of the cephalothorax and habitus. The first includes six genera with a rectangular cephalothorax and a cylindrical body shape. They are comprised of: *Arenosetella* Wilson, 1932; *Ectinosomoides* Nicholls, 1945; *Glabrotelson* Huys in Kihara & Huys, 2009; *Noodtiella* Wells, 1965; *Oikopus* Wells, 1967; and *Tetanopsis* Brady, 1910. The second group includes 15 genera with a cephalothorax tapering anteriorly and a fusiform body shape: *Bradya* Boeck, 1873; *Bradyellopsis* Brian, 1925; *Chaulionyx* Kihara & Huys, 2009; *Ectinosoma* Boeck, 1865; *Ectinosomella* Sars, 1910; *Halophytophilus* Brian, 1919; *Halectinosoma* Vervoort, 1962; *Klieosoma* Hicks & Schriever, 1985; *Microsetella* Brady & Robertson, 1873; *Parahalectinosoma* George & Schwabe, 2019; *Parabradya* Lang, 1944; *Pseudectinosoma* Kunz, 1935; *Pseudobradya* Sars, 1904; *Rangabradya* Karanovic & Pesce, 2001 (including *Siamorangabradya*, subgen. nov.); and *Sigmatidium* Giesbrecht, 1881. The third group comprises genera with a cephalothorax clearly wider than the urosome and a dorsoventrally depressed prosome, including *Peltobradya* Médioni & Soyer, 1968 and *Pontobradya* Apostolov, 2011. Of the above-mentioned genera, *Halectinosoma* is most speciose with 69 valid species (Sciberras et al. 2018).

The orientation of the maxillary allobasis is another significant taxonomic character, which can be divided into two types: prehensile and non-prehensile. The maxilla of most Ectinosomatidae is geniculated, with the allobasis considerably bending medially to form a prehensile appendage. In the other genera, as well as in the new species, the maxilla is not geniculate and an allobasis bends slightly, forming a slight angle between the syncoxa and the allobasis. A third group represented by the genus *Parahalectinosoma* has a maxilla that is strongly atrophied (George and Schwabe 2019).

Among the genera with a fusiform body, most characters of the Thai specimens match well the characteristics of *Rangabradya*. The shared characters are as follows:

1. the allobasis of the maxilla bends slightly, forming a wide angle with the syncoxa;
2. the mandibular gnathobase comprises a large convex space between the lacinia and pars incisiva;
3. the basal endite of the maxilla is situated in the first half-length of the syncoxa;
4. the allobasis of the maxilla is inserted by the medial seta at the middle of the segment;
5. P1–P4 Exp and Endp are three-segmented and the armature formula of P1–P4 Exp-3 is 5.6.6.6;
6. P5 in both sexes lacks a surface seta;
7. the female P5 Exp bears three spiniform setae and the outermost seta is the longest;
8. the male P5 Exp is completely fused to the baseoendopod;
9. the male P6 is reduced to a simple cuticular plate and has no armature element.

Of the nine shared characters, the combination of characters 6) and 7) confirms the close phylogenetic relationships between the new species from Thailand and the Indian species within the genus *Rangabradya*.

The female P5 in the new species is similar to that of *Ectinosoma* in that it has subdivided Exp and lacks a surface seta on the segment. However, the former has only three setae on Exp compared to the four reported for *Ectinosoma*. Three spiniform setae have also been observed in the female P5Exp in *Halectinosoma*, but its representatives have a surface seta on this segment.

Karanovic and Pesce (2001) suggested that *Rangabradya* is most similar to *Halectinosoma*, one of the primitive genera of the family Ectinosomatidae. Based on this point of view, several characters present in the new species are considered primitive compared to those of *R. indica* (Table 1). The new species probably represents one of the phylogenetic transitional stages between *Halectinosoma* and *Rangabradya*. However, because there is no significant character (i.e., apomorphic character) to distinguish the new species from the genus *Rangabradya*, we suggest establishing the new subgenus Siamorangabradya subgen. nov. to accommodate the new stygobiotic species, Rangabradya (Siamorangabradya) wongkamhaengae subgen. et sp. nov. from Thailand.

**Table 1.** Distinctive characters between Siamorangabradya subgen. nov. and the nominative subgenus Rangabradya.

Characters	Siamorangabradya subgen. nov.	* Rangabradya *
Ornamentation of urosomite	present	absent/reduced
Number of setae on caudal ramus	7	6
Number of segments of antennule	6	5
Spine between pars incisiva and lacinia on mandibular gnathobase	present	absent
Number of setae on mandibular Endp	8	5
Basal seta on praecoxal arthrite	present	absent
Setal formula of maxillary syncoxal endite from proximal to distal ones	3.2.3	4.1.2
Number of setae on maxillipedal Endp	4	3
Exp and baseoendopod of female P5	subdivided	fused
Number of setae on female P6	1	absent
Number of setae on endopodal lobe of the male P5	2	1

After Sciberras et al. (2018) the key to genera of Ectinosomatidae includes 22 known genera. The genus *Parahalectinosoma* George & Schwabe, 2019 was later described from the Kuril Basin, Russia (George and Schwabe 2019). It has a fusiform habitus, with numerous derived characters on its buccal appendages suggesting a symbiotic mode of life. As the Thai *Rangabradya* could not be identified using this key, the following amendments were made to the key in Sciberras et al. (2018: 420) by modifying the character detailed in paragraphs 13 and 14 and adding new couplets as the last paragraphs, as follows:

### Updated key to the genera of Ectinosomatidae

**Table:** 

1	Body cylindrical with cephalothorax rectangular in dorsal aspect; body approximately the same width throughout its length	**2**
–	Body fusiform with cephalothorax sub-triangular in dorsal aspect; greatest body width usually at posterior margin of cephalothorax; urosome gradually tapering towards the posterior end	**7**
–	Body with dorsoventrally depressed prosome, clearly wider than urosome	**20**
2	Exp of antenna two-segmented; maxilla prehensile, with major articulation between elongate syncoxa and elongate allobasis	***Noodtiella* Wells, 1965**
–	Exp of antenna one- or three-segmented; maxilla not prehensile, with at most a slight angle between syncoxa and allobasis	**3**
3	P2–P4Endp two-segmented	***Ectinosomoides* Nicholls, 1945**
–	P2–P4Endp three-segmented	**4**
4	Anal somite with dorsal armature of claws, lappets or spiniform processes around anal opening; P5Exp with three marginal and one surface seta	***Arenosetella* Wilson, 1932**
–	Anal somite without such ornamentation	**5**
5	Exp of antenna one-segmented	***Tetanopsis* Brady, 1910**
–	Exp of antenna three-segmented	**6**
6	Female P5Exp and baseoendopod with foliaceous setae, Exp with three marginal and no surface setae; male P5Exp with four normal marginal setae	***Oikopus* Wells, 1967**
–	P5Exp and baseoendopod with normal setae in both sexes, Exp with three marginal and typically a surface seta [absent in *Glabrotelson soyeri* (Bodin, 1979)]	***Glabrotelson* Huys in Kihara & Huys, 2009**
7	P1–P4Endp two-segmented	***Pseudectinosoma* Kunz, 1935**
–	P1Endp two- or three-segmented, P2–P4Endp three-segmented	**8**
8	P1Endp prehensile	**9**
–	P1Endp not prehensile	**12**
9	P1Endp two-segmented	**10**
–	P1Endp three-segmented	***Klieosoma* Hicks & Schriever, 1985**
10	P1–P2Exp-3 with two outer elements	**11**
–	P1–P2Exp-3 with three outer elements	***Halophytophilus* Brian, 1919**
11	Antennule with large spine on second segment (and often first and third segments); Exp of antenna rudimentary, with one to three small setae; P1Endp-2 with four elements (one to two pinnate and claw-like)	***Bradyellopsis* Brian, 1925**
–	Armature elements on first to third antennulary segments setiform; Exp of antenna well developed and three-segmented; P1Endp-2 with six elements (outer one bifid and claw-like)	***Chaulionyx* Kihara & Huys, 2009**
12	Maxilla prehensile, with syncoxa and allobasis forming right angle; P5Exp poorly developed, short, fused to baseoendopod in female and distinct in male, with three marginal and no surface setae; body very small (< 300 μm)	***Sigmatidium* Giesbrecht, 1881**
–	These characters not combined	**13**
13	Female P5Exp and baseoendopod fused, forming a single plate in both sexes, or partly discrete; male P6 forming a single plate or absent	**14**
–	Female P5Exp and baseoendopod at least partly discrete; male P6 with armature element	**15**
14	Armature formula of P1–P4Exp-3: 5, 6, 6, 6; male P6 absent or unarmed	**21**
–	Armature formula of P1–P4Exp-3: 6, 7, 8, 8; male P6 with two setae; body of female large (≥ 1200 μm); marine, usually deepwater	***Parabradya* Lang, 1944**
15	Integument of somites with distinctive sub-rectangular pores; P5Exp with four marginal setae	***Ectinosoma* Boeck, 1865**
–	Integument of somites without distinctive sub-rectangular pores; P5Exp with three marginal setae and one seta on anterior surface	**16**
16	Mandible with rudimentary gnathobase, elongate basis and filiform rami, each terminating in two to three setae; Exp of antenna without lateral spines	***Ectinosomella* Sars, 1910**
–	These characters not combined	**17**
17	Third segment of female antennule 3× as long as wide; Endp of mandible with one strong seta laterally; P1–P4Exp-3 with two outer spines; planktonic (occasionally in sediment)	***Microsetella* Brady & Robertson, 1873**
–	These characters not combined	**18**
18	Body comparatively robust with prosome–urosome separation usually distinct (exception: *Bradya kurtschminkei* Seifried & Martínez Arbizu, 2008 with dorsoventrally flattened habitus); antenna with two setae on Exp-1 and one seta on Endp-1; Exp of mandible with at least five setae; maxilliped robust with short Endp usually fused at an angle with basis and bearing four conspicuous setae	***Bradya* Boeck, 1873**
–	Body comparatively slender with no sharp separation between prosome and urosome; antenna with less than two setae on Exp-1 (except *Pseudobradya ambigua* Sars, 1920 with two) and no seta on Endp-1; Exp of mandible generally with less than five setae; maxilliped usually slender and straight with discrete Endp bearing one small and four conspicuous setae	**19**
19	Antennule with or without dark pigment spot within the proximal three segments; maxilla prehensile, allobasis usually truncate distally and carrying three-segmented Endp (although Endp sometimes very small and segmentation difficult to discern; reduced to a narrow three-segmented cylinder in *P. leptognatha* Sars, 1920); maxilliped short and robust	***Pseudobradya* Sars, 1904**
–	Antennule without pigment spot; maxilla with at most a slight angle between syncoxa and allobasis, the latter generally tapering distally, Endp three-segmented but always small, its morphology not clearly discernible; maxilliped generally slender	***Halectinosoma* Vervoort, 1962**
20	P1Endp three-segmented; female P5Exp with four marginal elements	***Pontobradya* Apostolov, 2011**
–	P1Endp two-segmented; female P5Exp with three marginal elements and one surface seta	***Peltobradya* Médioni & Soyer, 1968**
21	Exp of antenna three-segmented; maxillule and maxilla normally developed; maxilliped three-segmented; continental groundwater	***Rangabradya* Karanovic & Pesce, 2001**
–	Exp of antenna one-segmented; maxillule and maxilla strongly atrophied; maxilliped two-segmented; symbiosis in echiuran coelom	***Parahalectinosoma* George & Schwabe, 2019**
22	Female P5Exp fused to baseoendopod; male P5 with one seta on baseoendopodal lobe	**Rangabradya (Rangabradya) Karanovic & Pesce, 2001**
–	Female P5Exp and baseoendopod partly discrete; male P5 with two setae on baseoendopodal lobe	**Rangabradya (Siamorangabradya) subgen. nov.**

## Supplementary Material

XML Treatment for Siamorangabradya

XML Treatment for Rangabradya (Siamorangabradya) wongkamhaengae
